# An exploration of the missing data mechanism in an Internet based smoking cessation trial

**DOI:** 10.1186/1471-2288-12-157

**Published:** 2012-10-15

**Authors:** Dan Jackson, Dan Mason, Ian R White, Stephen Sutton

**Affiliations:** 1, MRC Biostatistics Unit, Cambridge, UK; 2 Behavioural Science Group, Institute of Public Health, University of Cambridge, Cambridge, UK

## Abstract

**Background:**

Missing outcome data are very common in smoking cessation trials. It is often assumed that all such missing data are from participants who have been unsuccessful in giving up smoking (“missing=smoking”). Here we use data from a recent Internet based smoking cessation trial in order to investigate which of a set of a priori chosen baseline variables are predictive of missingness, and the evidence for and against the “missing=smoking” assumption.

**Methods:**

We use a selection model, which models the probability that the outcome is observed given the outcome and other variables. The selection model includes a parameter for which zero indicates that the data are Missing at Random (MAR) and large values indicate “missing=smoking”. We examine the evidence for the predictive power of baseline variables in the context of a sensitivity analysis. We use data on the number and type of attempts made to obtain outcome data in order to estimate the association between smoking status and the missing data indicator.

**Results:**

We apply our methods to the iQuit smoking cessation trial data. From the sensitivity analysis, we obtain strong evidence that older participants are more likely to provide outcome data. The model for the number and type of attempts to obtain outcome data confirms that age is a good predictor of missing data. There is weak evidence from this model that participants who have successfully given up smoking are more likely to provide outcome data but this evidence does not support the “missing=smoking” assumption. The probability that participants with missing outcome data are not smoking at the end of the trial is estimated to be between 0.14 and 0.19.

**Conclusions:**

Those conducting smoking cessation trials, and wishing to perform an analysis that assumes the data are MAR, should collect and incorporate baseline variables into their models that are thought to be good predictors of missing data in order to make this assumption more plausible. However they should also consider the possibility of Missing Not at Random (MNAR) models that make or allow for less extreme assumptions than “missing=smoking”.

## Background

Missing outcome data are a very common problem in smoking cessation trials. It is common that any such missing data are assumed to correspond to smokers [1-4]. This assumption could be justified by the notion that anyone in a trial who successfully gives up smoking will report this fact. Foulds *et al*. [5] provide some evidence that missing data are smokers. Hajek and West [6] argue that the “missing=smoking” assumption is plausible because “successful quitters are usually keen to let the treatment providers know of their success” and that “treatment failures feel embarrassed”. The Russell standard requires that smokers lost to follow-up are classified as continuing to smoke [6,7].

However the evidence from Foulds *et al*. is of limited value because it is based upon just fifty participants with missing outcome data. Furthermore this was in a hospital setting and there is no reason why this should translate to other settings, and in particular to an Internet based trial. Although some may find the “missing=smoking” assumption plausible, and this provides a simple way to handle the missing data, it is open to immediate criticism. One reason for this is because imputing missing outcome data as smokers is a single imputation based procedure, which does not take into account the uncertainty in the missing values [8, p. 45]; if the “missing=smoking” assumption is incorrect then measures of uncertainty, such as standard errors, can be artificially and very considerably diminished. “Missing=smoking” also assumes that all quitters respond. Finally, the “missing=smoking” assumption tacitly assumes that any baseline and intermediate data have no additional value for predicting the outcome for participants whose outcome is unknown.

A further source of concern is the bias in the estimated treatment effect that may result from incorrectly assuming “missing=smoking”. Nelson *et al.*[9] show that this assumption is “as likely to lead to liberal estimates as to conservative estimates relative to the complete case analysis” and argue that better statistical methods are needed for handling missing data in the tobacco cessation research community. Barnes *et al.*[10] investigate a range of methods for handling missing data in their trial and conclude that imputing missing data as smokers “can cause a large amount of bias if imputing smoking is an incorrect assumption”.

The principal contribution of this paper is the use of empirical evidence to explore the plausibility of different missing data models in the context of a smoking cessation trial. Our aim is to determine which variables play an important role in these models. Particular interest lies in the role of the outcome itself, in order to assess the appropriateness of the “missing=smoking” assumption.

The rest of this paper is set out as follows. We begin by introducing the iQuit trial, an Internet based smoking cessation trial with a large amount of missing outcome data [11]. Here we also describe ten baseline covariates that were thought, a priori, to be potential predictors in the missing data model. We also describe the repeated attempts that were made by the trial investigators to obtain outcome data. In section “Which baseline variables are predictive of missingness? A selection model approach”, we develop our selection model, where we assess whether any of the baseline variables play an important role in the missing data model, whilst also allowing the outcome itself to influence this in a sensitivity analysis. The attempt to simultaneously estimate the baseline covariate and the outcome effects in this selection modelling framework was, as anticipated, not very successful so in section “Is the primary trial outcome predictive of missingness? Modelling the repeated attempts” we describe and use our model for the repeated attempts made to obtain outcome data. Finally we summarise our findings and draw conclusions for smoking cessation trialists.

## The iQuit trial

The iQuit trial is an Internet based smoking cessation randomised controlled trial to assess the benefit of self-help smoking cessation materials tailored to individual smoker characteristics over generic self-help materials, conducted among the general population of smokers seeking help from web-based sources [11]. Participants sign up for the trial via the QUIT website (www.quit.org.uk). They fill in a questionnaire and receive an online advice report to help them quit smoking. They are randomised either to receive the tailored version or the generic version. Six months later they receive a telephone interview to find out whether they are still smoking, see if their smoking-related beliefs have changed at all, and find out what they thought of the advice they received.

The primary iQuit trial outcome is whether or not participants have abstained from smoking (self-reported three months prolonged abstinence) and the primary research question is whether or not tailored materials are more effective than generic materials in helping participants achieving this. The corresponding analysis is described in detail by Mason *et al.*[11], who found a lack of evidence for a treatment effect. However, and despite the intensive follow up from the trialists to obtain outcome data, there is a large amount of missing data; smoking status is unknown for 1036 of the 1758 participants (59%). This compromises the primary analysis, as explained by Mason *et al.*, but provides an excellent opportunity to investigate the reasons for missing data. The pattern of missing data is summarised in Table 1.

**Table 1:** The pattern of missingness for the primary trial outcome *Y*

	**Treatment**	**Control**	**Total**
	**(tailored)**	**(generic)**	
Not abstained (*Y* = 0)	271	289	560
Abstained (*Y* = 1)	80	82	162
Missing *Y*	526	510	1036
Total	877	881	1758

In addition to the primary trial outcome, a wide range of complete (no missing data) baseline variables were measured, and those thought a priori to be most likely to be good predictors of missing data are summarised in Table 2. Some of the variables in Table 2 are referred to as ‘smoking related’ variables because they are considered to more directly relate to the participant’s smoking behaviour. An aim here is to investigate which, if any, of these variables are good predictors, whilst allowing for the possibility that the primary trial outcome itself may also be predictive of its missingness.

**Table 2:** Baseline covariates

**Covariate**	**Parameter**	**Type**	**Smoking related?**	**Description**	**Summary statistics**
Treatment	*β*_1,1_	Binary	No	Indicator for treatment group	877/1758 treated
Age	*β*_1,2_	Continuous	No	Age in years	38 (11)
Sex	*β*_1,3_	Binary	No	Indicator for a female participant	1126/1758 female
Qualifications	*β*_1,4_	Categorical	No	Educational qualifications: 1=None; 2=GCSE	3.06 (1.16)
				3=A-level; 4=Undergraduate Degree; 5=Postgraduate Degree	
Deprivation	*β*_1,5_	Categorical	No	Deprivation score, range 0-5, higher indicates more deprived	1.21 (1.09)
Conscientiousness	*β*_1,6_	Continuous	No	Conscientiousness score, range 1-5, higher	3.31 (0.84)
				indicates more conscientiousness (takes values between 1-5, in steps of 0.25, and so is not truly continuous).	
Determination	*β*_1,7_	Categorical	Yes	Determination to quit: 1=not at all; 5=extremely	4.30 (0.75)
Support	*β*_1,8_	Categorical	Yes	Does the participant feel supported by family and friends: 1=not at all; 5=extremely	3.31 (1.23)
Dependence	*β*_1,9_	Categorical	Yes	Cigarette dependence score, range 1-8,	5.48 (1.57)
				higher indicates greater dependence	
Previous	*β*_1,10_	Binary	Yes	Indicator for not having managed to quit previously	907/1758 have

Summary statistics are shown where means and standard deviations (in parentheses) are given for the continuous and categorical variables.

The iQuit trial provides the rich data on the number and type of attempts made to obtain outcome data shown in Table 3. It was specified in the trial design that participants would receive up to ten telephone calls to obtain outcome data and, for those of whom all calls were unsuccessful, where possible a single further attempt was made by email. The telephone attempts ceased when outcome data was obtained, the number given by the participant was found to be invalid or the participant requested that no further telephone calls were made. The decision to make telephone calls to obtain outcome data was made in order to ensure good quality data and so that the medium of follow-up was not the same as the medium of intervention. Multiple telephone calls were made in order to facilitate calling participants at different times of the day but no more than ten calls were made to avoid harassing them. The email was a ‘last ditch’ effort to obtain outcome data where the telephone calls had failed. These repeated attempts to obtain data provide the basis for our modelling in section “Is the primary trial outcome predictiveof missingness? Modelling the repeated attempts”.

**Table 3:** The outcome *Y * by the number of contact attempts and trial arm

**Attempts**	**Participants**	**Responded**	**Of responders % quit**
One phone call	217	214	50/214=23.4%
One phone call and an email	925	69	30/69 =43.5%
Two phone calls	134	132	23/132=17.4%
Two phone calls and an email	6	1	0/1=0%
Three phone calls	93	89	16/89=18.0%
Three phone calls and an email	3	0	0/0
Four phone calls	59	57	10/57=17.5%
Four phone calls and an email	4	0	0/0
Five phone calls	48	45	9/45=20.0%
Five phone calls and an email	8	0	0/0
Six phone calls	19	19	1/19=5.3%
Six phone calls and an email	2	1	1/1=100%
Seven phone calls	38	38	5/38=13.2%
Seven phone calls and an email	2	0	0/0
Eight phone calls	17	16	3/16=18.8%
Eight phone calls and an email	5	1	1/1=100%
Nine phone calls	11	10	2/10=20%
Nine phone calls and an email	2	0	0/0
Ten phone calls	19	19	6/19=31.6%
Ten phone calls and an email	146	11	5/11=45.5%

The fraction and percentage of participants who successfully quit smoking (Y=1) are tabulated by the number of contact attempts (telephone calls and email). Participants received up to ten telephone calls and up to one email attempt.

## Which baseline variables are predictive of missingness? A selection model approach

Here a selection modelling approach [8, p. 30] is used in order to investigate the missing data model in smoking cessation trials. The modelling allows for an association between the trial outcome and the missing data indicator but also accommodates less extreme assumptions than “missing=smoking”. We extend this approach in the next section by using data on the repeated attempts to obtain outcome data [12-15].

For the sake of generality, for the moment we use vectors to denote the outcomes but in our application these quantities are scalars. Let **Y**_*i*_denote the *i*th participant’s vector of outcomes, so that participants may provide more than a single outcome, and let **R**_*i*_ denote the corresponding vector of missing data indicators, where *R*_*i*,*j*_ = 1 if *Y*_*i*,*j*_ is observed, where *Y*_*i*,*j*_ and *R*_*i*,*j*_ are the *j*th entries of **Y**_*i*_ and **R**_*i*_ respectively. We let **x**_*i*_ denote the *i*th participant’s covariates and we posit a model for **Y**_*i*_| **x**_*i*_. We then posit a model **R**_*i*_|( **Y**_*i*_, **x**_*i*_), which is referred to as the selection model. We model the joint distribution of ( **Y**_*i*_, **R**_*i*_)| **x**_*i*_ using the factorisation provided by these two models.

A common assumption is that the data are Missing at Random (MAR). The data are said to be MAR, given the covariates **x**_*i*_ if, for all *i*, **R**_*i*_ is independent of the missing entries of **Y**_*i*_, given those that are observed and **x**_*i*_. Equivalently, the MAR assumption can be expressed as the requirement that the density of **R**_*i*_|( **Y**_*i*_**x**_*i*_) depends only on **Y**_*i*_through the entries that are observed. However it is not clear from this definition whether MAR requires this condition for the observed pattern of missing data or for all possible patterns of missing data under repeated sampling. The definition of MAR of Lu and Copas [16] makes this requirement explicit for all possible missingness patterns and they show that, with the further assumption that separate parameters are used in the models for the outcome and the selection model, their definition of MAR implies that the model for the missing data **R**_*i*_|( **Y**_*i*_**x**_*i*_) is ignorable and valid inferences for the outcome parameters can be made using just the outcome model and the observed outcome data. A caveat however is that the observed, rather than the expected, information matrix should be used to obtain standard errors [17].

If the MAR assumption is not satisfied then the data are Missing not at random (MNAR). We will allow MNAR models so that an association between the potentially missing outcome (smoking cessation) and the missing indicator is permitted. Although the model for the outcome is usually of central interest, because this contains population parameters such as a treatment effect, here the focus of interest lies in the selection model. This is because this model describes *why* data are missing, and so we also refer to this model as the missing data model.

We are primarily interested in determining which variables play an important role in this model. One reason why this investigation is important is because MAR analyses are made more plausible by including variables that are good predictors of missingness: if they predict missingness sufficiently well so that any role of missing **Y**_*i*_ is non-existent, or at least negligible, then the MAR assumption is adequate. It is however important to know what kind of additional variables smoking cessation trialists should routinely collect and incorporate into models to make MAR more plausible. These variables may be modelled as covariates if we are prepared to adjust for them [18], or as further response variables if we are not [18,19]. Another reason why this investigation is important is to determine whether or not the outcome itself is a useful predictor of missingness, in order to assess whether MNAR modelling is required. However, since every MNAR model has a MAR counterpart with equal fit [20], it is only by making distributional assumptions, such as those that follow, that this type of assessment can be made.

We will define *Y*_*i*_ = 1 if the *i*th participant has abstained from smoking and *Y*_*i*_ = 0 otherwise and *R*_*i*_ as the corresponding missing data indicator. We define **x**_*i*_as the *i*th participant’s row vector of ten covariates, in the order they appear in Tables 2 and 4. Since *Y*_*i*_ and *R*_*i*_ are both binary, we use conventional logistic regression modelling for both variables and we assume that

(1) $$\text{logit}(P(Y_{i}=1|x_{i}))=\alpha_{0}+\alpha_{1}x_{i}$$

and

(2) $$\text{logit}(P(R_{i}=1|Y_{i}=y_{i},x_{i}))=\beta_{0}+\beta_{1}x_{i}+\beta_{2}y_{i}.$$

**Table 4:** The results from the sensitivity analysis

**Parameter**	***β***_**2**_*** = − 4***	***β***_**2**_*** = − 3***	***β***_**2**_*** = − 2***	***β***_**2**_*** = − 1***	***β***_**2**_*** = 0***	***β***_**2**_*** = 1***	***β***_**2**_*** = 2***	***β***_**2**_*** = 3***	***β***_**2**_*** = 4***
	( ***P***_***a***_*** = 0.94***)	( ***P***_***a***_*** = 0.85***)	( ***P***_***a***_*** = 0.68***)	( ***P***_***a***_*** = 0.44***)	( ***P***_***a***_*** = 0.22***)	( ***P***_***a***_*** = 0.10***)	( ***P***_***a***_*** = 0.04***)	( ***P***_***a***_*** = 0.01***)	( ***P***_***a***_*** = 0.00***)
*β*_1,1_ (Treatment)	-0.073(0.163)	-0.072(0.149)	-0.081(0.127)	-0.088(0.106)	-0.091(0.099)	-0.094(0.102)	-0.095(0.104)	-0.097(0.106)	-0.097(0.107)
*β*_1,2_ (Age)	**0.038(0.008)**	**0.035(0.007)**	**0.033(0.006)**	**0.030(0.005)**	**0.028(0.005)**	**0.027(0.005)**	**0.026(0.005)**	**0.026(0.005)**	**0.025(0.005)**
*β*_1,3_ (Sex)	0.005(0.172)	0.036(0.157)	0.070(0.133)	0.102(0.111)	0.131(0.104)	0.152(0.107)	0.165(0.110)	0.171(0.111)	0.174(0.112)
*β*_1,4_ (Qualifications)	0.072(0.077)	0.073(0.070)	0.072(0.061)	0.071(0.051)	0.072(0.047)	0.073(0.049)	0.071(0.050)	0.069(0.050)	0.067(0.050)
*β*_1,5_ (Deprivation)	**-0.253(0.088)**	**-0.222(0.079)**	**-0.175(0.067)**	**-0.116(0.055)**	-0.066 (0.051)	-0.036(0.053)	-0.024(0.054)	-0.020(0.054)	-0.018(0.054)
*β*_1,6_ (Conscientiousness)	**-0.230(0.097)**	**-0.208(0.089)**	**-0.177(0.076)**	**-0.139(0.064)**	-0.105(0.060)	-0.084(0.062)	-0.074(0.063)	-0.069(0.064)	-0.067(0.064)
*β*_1,7_ (Determination)	**0.240(0.114)**	**0.206(0.103)**	0.153(0.087)	0.089(0.073)	0.034(0.067)	0.001(0.069)	-0.015(0.071)	-0.022(0.071)	-0.024(0.072)
*β*_1,8_ (Support)	0.131(0.067)	**0.121(0.062)**	**0.107(0.052)**	**0.087(0.044)**	0.069(0.041)	0.057(0.042)	0.051(0.043)	0.049(0.044)	0.048(0.044)
*β*_1,9_ (Dependence)	0.059(0.059)	0.055(0.052)	0.039(0.043)	0.016(0.036)	-0.001(0.034)	-0.011(0.035)	-0.015(0.035)	-0.017(0.036)	-0.018(0.036)
*β*_1,10_ (Previous)	-0.020(0.169)	-0.041(0.153)	-0.084(0.131)	-0.138(0.109)	-0.183(0.103)	**-0.211(0.105)**	**-0.224(0.108)**	**-0.228(0.109)**	**-0.230(0.110)**

The coefficients *β*_1,1_ to *β*_1,10_ describe the effect of each of the ten baseline covariates in Table 2. The tabulated *P*_*a*_ = *P*(*Y* = 1|*R* = 0,**x**) are obtained from equation (4) with logit(*P*(*Y* = 1|*R* = 1,**x**)) = logit(0.22)and the corresponding value of *β*_2_. Statistically significant estimates, at the 5% level, are shown in bold and standard errors are in parentheses.

If *R*_*i*_ = 0 then *Y*_*i*_ is missing and is ‘summed out’ of the log-likelihood in (3) below. Hence participants who do not provide outcome data contribute to the analysis. We further assume that participants are independent. The first *α*_1_ parameter, which we denote as *α*_1,1_ is the (adjusted) treatment effect, but here the focus of interest is on the covariates that are important in the missing data model, ie *β*_1_ and *β*_2_ are paramount. The parameter *β*_2_ is the adjusted log odds ratio between *Y*_*i*_and *R*_*i*_. This parameter is therefore of particular interest because a positive infinite *β*_2_ is equivalent to assuming “missing=smoking”. If *β*_2_ = 0 then the data are MAR, otherwise the data are MNAR. We address the difficulty in estimating *β*_2_ later. Separate parameters are used for the outcome (*α* parameters) and the selection model (the *β*parameters) so MAR implies that the missing data model is ignorable [16]. In this case the models (1) and (2) can be fitted as two separate conventional logistic regressions, where model (1) is fitted using the complete cases.

A participant for whom *Y*_*i*_is observed ( *R*_*i*_ = 1) contributes *P*( *Y*_*i*_ = *y*_*i*_| **x**_*i*_)*P*( *R*_*i*_ = 1| *Y*_*i*_ = *y*_*i*_, **x**_*i*_) to the likelihood, and a participant for whom *Y*_*i*_ is not observed provides $P(R_{i}=0|x_{i})=\underset{y_{i}}{\sum }P(Y_{i}=y_{i}|x_{i})P(R_{i}=0|Y_{i}=y_{i},x_{i})$. The log-likelihood of the data provided by all 1758 participants is *L*( *α*_0_, *α*_1_, *β*_0_, *β*_1_, *β*_2_) = 

(3) $$\begin{array}{lll} & \;\overset{1758}{\underset{i=1}{\sum }}R_{i}\log\left\{P(Y_{i}=y_{i}|x_{i})P(R_{i}=1|Y_{i}=y_{i},x_{i})\right\}\; & \;\\ & \;+\overset{1758}{\underset{i=1}{\sum }}(1-R_{i})\log\left\{P(R_{i}=0|x_{i})\right\}\; & \;\\ & \;=\overset{1758}{\underset{i=1}{\sum }}R_{i}\log\left\{P(Y_{i}=y_{i}|x_{i})P(R_{i}=1|Y_{i}=y_{i},x_{i})\right\}\; & \;\\ & \;+\overset{1758}{\underset{i=1}{\sum }}(1-R_{i})\log\left\{\overset{1}{\underset{y_{i}=0}{\sum }}P(Y_{i}=y_{i}|x_{i})P(R_{i}=0|Y_{i}=y_{i},x_{i})\right\}\; \end{array}$$

where the probabilities necessary to compute this likelihood are evaluated in terms of the *α* and *β* parameters from equations (1) and (2). Participants who provide outcome data ( *R*_*i*_ = 1) contribute to the first summation in (3) and those who do not provide outcome data ( *R*_*i*_ = 0) contribute to the second summation.

### Modelling the covariates

Complete case logistic regressions (analyses that assume MAR) were performed for the outcomes *Y * on each of the categorical variables in Table 2 in turn, where the regressions treated these variables as categorical and then continuous. Deviance tests (comparing the fitted logistic regressions treating these variables as categorical and continuous), suggested that treating the categorical variables as continuous in the model (1) is adequate. Similar results were obtained for regressions of the missing data indicator, providing reassurance that treating these variables as continuous in (2) is also adequate.

In situations where the treatment of categorical variables as continuous does not appear so reasonable, two approaches might be considered. First the categorical variables could be treated as such, but the additional dummy variables will make the already computationally demanding nature of MNAR modelling yet more so. An alternative is to dichotomise categorical variables, where care is taken to ensure that there is a reasonable amount of data in both groups and, ideally, the sensitivity of the results to the decisions made when dichotomising variables is assessed. A limitation of our investigations of the treatment of the categorical variables as continuous is that these are from standard logistic regressions, which assume data are MAR, but the computationally intensive nature of using the full likelihood very much reduced the appeal of using MNAR models in preliminary investigations of this kind.

We also investigated the possibility that quadratic terms for the two continuous covariates in Table 2 might be required in (1) and (2); no evidence was found that these are required to describe the data. More sophisticated transformations of continuous covariates, for example using spline functions or fractional polynomials, could also be considered but these would add to the computational demands and were not explored. As a final point, interactions between the ten covariates could be considered. The introduction of further parameters to the likelihood also adds to the computational demands and this was not investigated, in part because of this, but also because we merely wish to assess which covariates present themselves as important predictors in model (2), for which our modelling is adequate.

### A sensitivity analysis

The estimation of the full selection model using the log-likelihood (3) is generally discouraged because the model fit is so fragile; it is highly dependent on distributional assumptions and is sensitive to outlying or unusual observations [21]. Sensitivity analyses are therefore generally encouraged and so we adopt this approach in this section, where *β*_2_ is used as the sensitivity parameter. We know that *β*_2_ = 0(MAR) generally provides a stable model fit so we anticipate that this will also be so for alternative fixed values of *β*_2_. However this can only assess baseline covariate effects assuming particular values for the sensitivity parameter, and cannot quantify the evidence that the outcome itself is important in the missing data model. We return to the estimation of the full selection model in section “Fitting the full model’. For the moment we are content to address the question of which baseline variables play an important role in the missing data model.

In our sensitivity analysis, we constrain *β*_2_, to nine values: -4, -3, -2, -1, 0 , 1, 2, 3, 4. The values *β*_2_ were chosen because they cover a wide range of possibilities. This can be seen by noting that

(4) $$\;\beta_{2}=\text{logit}(P(R_{i}\;=\;1|Y_{i}\;=\;1,x_{i}))-\text{logit}(P(R_{i}\;=\;1|Y_{i}\;=\;0,x_{i}))$$

which, because the odds ratio treats the two variables being compared symmetrically, is equivalent to

(5) $$\begin{array}{lll} \beta_{2}= & -(\text{logit}(P(Y_{i}=1|R_{i}=0,x_{i}))\; & \;\\ & -\text{logit}(P(Y_{i}=1|R_{i}=1,x_{i})))=-\log(\text{IMOR})\; \end{array}$$

where ‘IMOR’ is the Informatively Missing Odds Ratio of Higgins *et al*[22]. We take the covariates **x**_*i*_ as referring to a typical participant who has *P*(*Y* = 1|*R* = 1,**x**)equal to the observed abstention rate in the complete cases, ie *P*(*Y* = 1|*R* = 1,**x**) = 162/722≈0.22. We can then approximately convert *β*_2_ values to *P*( *Y*_*i*_ = 1| *R*_*i*_ = 0, **x**_*i*_), using equation (4). This approximate conversion from *β*_2_ to *P*(*Y* = 1|*R*= 0,**x**) gives the values shown in Table 4, where we see that *β*_2_ = −4corresponds to a 94% abstention rate in (typical) participants with missing data, which is implausibly large, and *β*_2_ = 4corresponds to less than a 0.5% abstention rate which is tantamount to assuming “missing=smoking”. Hence the sensitivity analysis explores a very wide range of possibilities.

As explained above, the MAR model ( *β*_2_ = 0) can easily be fitted as separate logistic regressions. The remaining eight models are fitted by numerically maximising the log-likelihood (3), where parameter estimates’ standard errors are obtained from the observed information matrix, which is also obtained numerically. The log-likelihood was coded in R and the *maxLik* package was used to obtain the maximum likelihood estimates and their standard errors in this way. Starting values are required by the *maxLik* command and the MAR fit was used as starting values for *β*_2_ = − 1 and *β*_2_ = 1, and the resulting estimates were used as starting values for *β*_2_ = −2and *β*_2_ = 2, and so on. Despite this, several hours of computing time was needed to fit each of the eight models. The iQuit data are not freely available but indicative R code is available from the first author on request.

From Table 4 we have very robust inferences that age is an important predictor in the missing data model; no matter what value we assume for *β*_2_ we obtain strong evidence that older participants are more likely to provide outcome data. Evidence, at the 5% level, that the nonsmoking related variables ‘deprivation’ and ‘conscientiousness’ are important predictors requires negative *β*_2_, which means that participants with missing data are more likely to have given up smoking than those who provide outcome data. Those who consider “missing=smoking” plausible are unlikely to entertain negative *β*_2_ but such values might be justified by assuming that participants who have given up smoking are more likely to lose contact with the trial, because they no longer need its support, and so are in fact less likely to provide outcome data. Even if this possibility were entertained, it is clear that the significance of all non-smoking related covariate effects, other than the effect of age, are sensitive to *β*_2_ and hence the assumed role of the outcome in the selection model.

From Table 4, the significance of the smoking related covariates are also sensitive to the assumed value of *β*_2_; although some analyses provide significant effects, no covariate effect can be found at the 5% level that is not sensitive to the assumed *β*_2_. Only by making strong assumptions about the value of *β*_2_ can covariate effects be inferred.

To summarise the conclusions from the sensitivity analysis, the only baseline covariate that appears to be safely regarded as important in the missing data model is the age of participants. However other variables may also be important, depending on range of *β*_2_ thought plausible. 

### Fitting the full model

Despite our reservations about the full MNAR model fit being so fragile, we also fitted this model by numerically maximising the log-likelihood (3). This was achieved by using the MAR model fit as the starting point for the numerical maximisation and the resulting estimated missing data model is shown in Table 5. This model is (very weakly) identified by the assumptions made in the linear predictor in model (2). A saturated logistic regression model for the situation where data were available for all four combinations of outcome and missing data indicator is not identifiable here, because we do not observe data where the missing data indicator is 0. Hence the model identification must come from the form of model (2), which assumes linearity and no interactions.

**Table 5:** Estimates from the full selection model

**Parameter**	**Estimate**	**Standard error**
*β*_1,1_ (treatment)	-0.095	0.103
*β*_1,2_ (age)	0.026	0.006
*β*_1,3_ (sex)	0.160	0.118
*β*_1,4_ (qualifications)	0.072	0.049
*β*_1,5_ (deprivation)	-0.028	0.069
*β*_1,6_ (conscientiousness)	-0.077	0.073
*β*_1,7_(determination)	-0.010	0.084
*β*_1,8_(support)	0.053	0.046
*β*_1,9_(dependence)	-0.014	0.037
*β*_1,10_(previous)	-0.219	0.114
*β*_2_ (Y)	1.561	3.535

Despite our reservations, and the criticisms in literature of attempting this, the full MNAR selection model is identifiable and resulted in the following parameter estimates. See section Fitting the full model for a discussion of the difficulties in fitting the MNAR model.

A comparison of the MAR *β*_1_ estimates in Table 4 with the corresponding MNAR estimates in Table 5 suggests these are not very sensitive to the choice between assuming MAR or allowing this form of MNAR. The effect of age is again strongly significant, providing further weight to the evidence that this plays an important role in the missing data model. The standard errors of the *β*_1_ parameters increase slightly when allowing MNAR, but not as much as might be anticipated from the uncertainty in the estimate of *β*_2_ in Table 5; a 95% confidence interval for this parameter is (-5.4, 8.5) which includes all of the possibilities considered in the sensitivity analysis. Again making use of (4), the lower and upper bounds of the 95% confidence interval for *β*_2_ are close to “missing=cessation” and “missing=smoking” respectively so it it not possible to make any statement about the plausibility, or otherwise, of the commonly made ‘missing=smoking” assumption from this analysis. In any case, even if the standard error of *β*_2_ had been much smaller, any conclusions about the role of the outcome would be open to criticism due to issues surrounding the fitting of MNAR models [21].

## Is the primary trial outcome predictive of missingness? Modelling the repeated attempts

In order to overcome the problems associated with the estimation of MNAR missing data models using selection models, models for the repeated attempts to obtain outcome data have been proposed [12-15]. This type of modelling is possible where a number of attempts to obtain outcome data are made, as is the case for the iQuit trial: as explained above, participants in the iQuit trial receive between one and ten telephone calls to obtain outcome data, and if these are unsuccessful they receive where possible a further attempt by email. Participants may receive less than ten telephone calls and then an email if, for example, they request that no more telephone calls are made and do not provide data, or if the telephone number they have provided is found to be invalid. The assumption that underlies the modelling is that outcome data from participants who require many attempts to obtain are more like those with missing data than those who require fewer attempts.

We now model the probability that a particular attempt at obtaining outcome data is successful, rather than the marginal probability that outcome data is obtained as in selection modelling. We continue to assume model (1) and we replace model (2) with our model for the attempts to obtain outcome data

(6) $$\text{logit}(P(R_{i,m}^{\prime }=1|Y_{i}=y_{i},x_{i})=\beta_{0,m}+\beta_{1}x_{i}+\beta_{2}y_{i}$$

where $R_{i,m}^{\prime }$ is equal to one if the *m*th attempt, *m* = 1,2⋯11, to obtain outcome data from the *i* participant is successful; the email attempt is modelled as the 11th attempt regardless of the number of telephone calls made. We allow the email attempt to be more or less successful than the telephone attempts via its intercept *β*_0,11_ but make the simplification that the probability of obtaining outcome data in this way does not depend on the number of telephone calls that preceded it. This is reasonable because the email is a very different way to obtain outcome data and and this represents a pragmatic approach to modelling because the email was not very successful (only 83 participants provided data in response to over a thousand emails). This assumption is relaxed as part of the sensitivity analysis below. Model (5) can be thought of as a discrete survival model, or a stratified logistic regression, where we also handle the unobserved outcomes.

Each attempt has its own intercept *β*_0,*m*_, so that, for example, earlier attempts may be more successful than later ones but the identifying assumption is that the covariate effects are common across attempts. The appropriateness of this assumption for the baseline covariates was assessed by including an attempt by covariate interaction in the MAR model. Two of these interactions were statistically significant at the 5% level (age, p-value=0.04; qualifications, p-value=0.01). On a closer examination the apparent interaction between attempt and qualifications is largely explained by the observation that more educated participants appear to be more likely to respond to the email. Adding an interaction between the email attempt alone and the baseline covariates resulted in only one statistically significant interaction at the 5% level: the test for the presence of a qualifications by email interaction provided a p-value of 0.0004, where the log odds ratio associated with a unit increase in educational qualifications is 0.38. This may be plausible, because more educated participants could have greater access to, and command of, computing facilities. However, since a very small proportion of email attempts were successful, this finding should be cautiously interpreted. Despite this, more sophisticated modelling of the missing data model could commence by allowing this interaction.

Now that model (5) has replaced model (2), we refer to model (5) as the missing data model. If a single attempt is made to obtain outcome data from all participants then model (5) simplifies to (2), hence the model for the repeated attempts is an extension of the selection model.

The numbers and percentages of participants who successfully give up smoking ( *Y*_*i*_ = 1) are shown by the number and type of attempts to obtain outcome data in Table 3. Finding patterns in the results for those who do not respond the telephone calls and hence are sent an email is difficult, because such little outcome data is obtained in this way, but the data for those who respond to a telephone call is slightly suggestive of a decreasing probability of smoking cessation as the number of attempts increases: fitting a complete case logistic regression of smoking cessation on the number of attempts for these participants gives an estimated slope of -0.03 (with a standard error of 0.04). Although not significant, the fitted model predicts that the probability of smoking cessation decreases with the number of attempts. Therefore we anticipate a positive estimated association between *Y*_*i*_ and *R*_*i*,*m*_ when fitting model (5), so that those who have given up smoking are more likely to provide outcome data than those who have not.

The proportion of those giving up smoking is higher in those who respond to the email rather than a telephone call in Table 3. This could be because this method for obtaining outcome data, although less likely to obtain data per se, is relatively more likely to obtain outcome data from nonsmokers than smokers. If true this would invalidate the assumption that the *β*_2_ coefficient is the same for the email as the telephone attempts. Since estimating a separate *β*_2_ for the email attempt would encounter the same type of estimation problems as in section “Fitting the full model”, an alternative would be to constrain the *β*_2_ for the email attempt to a range of plausible values in a further sensitivity analysis.

The likelihood is similar in form to (3) but, now that each attempt to obtain outcome data contributes to this, its form is more complex and is shown in the Appendix. The MAR model ( *β*_2_ = 0) was fitted as separate logistic regressions of *Y*_*i*_on **x**_*i*_, and *R*_*i*,*j*_ on **x**_*i*_ and attempt number, in the same manner as for the MAR model in the sensitivity analysis above. This MAR model was then used as a starting value for the numerical maximisation of the full log-likelihood and standard errors can be obtained from the observed information matrix as before.

The fitted MAR and MNAR repeated attempts models are shown in Table 6. The estimates of the *β*_1_ parameters are not sensitive to the choice between MAR or MNAR and only slightly larger standard errors for these parameters are obtained when allowing the data to be MNAR. From Table 6 we see that the participant’s age is confirmed as having an important role in the missing data model and the effect of support (of family and friends) is also statistically significant, where participants who feel more supported are also more likely to provide outcome data. This analysis suggests that both smoking related (support) and non-smoking related (age) variables are good predictors of missing data so it would appear that both types of variables may play important roles in the missing data model.

**Table 6:** Estimates from the model for the repeated attempts

**Parameter**	**MAR estimate**	**MAR standard error**	**MNAR estimate**	**MNAR standard error**
*β*_1,1_ (treatment)	-0.099	0.081	-0.103	0.081
*β*_1,2_ (age)	0.021	0.004	0.021	0.004
*β*_1,3_ (sex)	0.103	0.086	0.110	0.087
*β*_1,4_ (qualifications)	0.034	0.040	0.033	0.040
*β*_1,5_ (deprivation)	-0.026	0.043	-0.018	0.044
*β*_1,6_ (conscientiousness)	-0.009	0.050	-0.001	0.050
*β*_1,7_ (determination)	-0.002	0.055	-0.008	0.055
*β*_1,8_ (support)	0.096	0.034	0.093	0.034
*β*_1,9_ (dependence)	0.020	0.027	0.018	0.028
*β*_1,10_ (previous)	-0.072	0.085	-0.076	0.085
*β*_2_ (Y)	-	-	0.215	0.222

The model for the repeated attempts incorporates more data, and hence makes more assumptions, but provides much more satisfactory estimation of *β*_2_, and hence the role of the outcome *Y* in the missing data model, than the selection model.

The model for the repeated attempts has enabled us to identify the effect of the trial outcome in the missing data model because the standard error of ${\hat{\beta }}_{2}$ is acceptably small. This is in sharp contrast with the results for the corresponding results using the selection model in section “Fitting the full model”. The estimate of *β*_2_ is positive as expected but the analysis, which rests on the distributional assumptions described above, does not rule out the possibility that data are MAR because *β*_2_ = 0 lies within the 95% confidence interval. We next explore what probabilities are predicted for smoking cessation in the missing data.

### A guide to the probability of abstaining from smoking for participants who do not provide outcome data

Let {*R*^*′*^}_*i*_ denote the set of $R_{i,m}^{\prime }$ observed for participant *i*; for example if a participant receives two telephone calls and an email then their ${\left\{R^{\prime }\right\}}_{i}=\{R_{i,1}^{\prime },R_{i,2}^{\prime },R_{i,11}^{\prime }\}$. For such a participant with missing data {*R*^*′*^}_*i*_ = {0,0,0} = {0}. From Bayes’ Theorem we obtain the odds that participants with missing data are smokers, given the failed attempts to obtain outcome data, as

(7) $$\begin{array}{lll} \frac{P(Y_{i}=1|{\left\{R^{\prime }\right\}}_{i}=\{0\},x_{i})}{P(Y_{i}=0|{\left\{R^{\prime }\right\}}_{i}=\{0\},x_{i})}= & \left\{\frac{P(Y_{i}=1|x_{i})}{P(Y_{i}=0|x_{i})}\right\}\; & \;\\ & \times \left\{\frac{P({\left\{R^{\prime }\right\}}_{i}=\{0\}|Y_{1}=1,x_{i})}{P({\left\{R^{\prime }\right\}}_{i}=\{0\}|Y_{1}=0,x_{i})}\right\}\; \end{array}$$

The terms in the first curly bracket on the right hand side of (6) can be obtained from model (1) and the probabilities that each of the $R_{i,m}^{\prime }$ that are members of {*R*^*′*^}_*i*_are zero can be obtained from model (5). Hence the odds, and therefore the probability, of not smoking given the failed attempts can be evaluated for participants with missing data. When fitting the full model using maximum likelihood, models (1) and (5) are fitted simultaneously. Using the MNAR maximum likelihood estimates to evaluate *P*( *Y*_*i*_ = 1|{*R*^*′*^}_*i*_ = {0},_**x***i*_) for all participants with missing data, and taking the average, gives a marginal probability of participants with missing data being nonsmokers of 0.17; 162/722=22% of those with observed outcomes are nonsmokers (Table 1). This analysis suggests that fewer participants with missing data are nonsmokers but does not support the “missing=smoking” assumption. For comparison, using the maximum likelihood estimates but replacing *β*_2_ with a value two standard errors above and below its estimate provides a marginal probability of participants with missing data being nonsmokers of 0.14 and 0.19 respectively; using *β*_2_ = 0 (MAR) in this way gives a probability of 0.18. These smaller probabilities of participants being nonsmokers, than in the sample of participants who provide outcome data, are partly due to them having covariates that are associated with less chance of giving up smoking but this probability also falls as *β*_2_ increases. Hence the choice of covariates that are included in the modelling affects the proportions of non-responders that are ‘imputed’ as smokers by the model.

This more sophisticated method for translating *β*_2_ into the probability that non-responders have abstained, which takes into account covariate effects, could also be used in conjunction with the selection model, but the approach adopted there is considerably simpler and more transparent.

### Further sensitivity and subgroup analyses

Since the assertion that the data do not support the “missing=smoking” assumption is such an important conclusion, we performed sensitivity analyses in order to assess how robust this inference is. First, we refitted the model including only the smoking related covariates (Table 2), then only the nonsmoking related covariates and then omitting all covariates.

Next we performed our subgroup analyses by fitting the full model to participants of median age (36) or under, and then to the older participants. We then fitted the full model to men and women separately, but omitting the now unidentifiable effect of sex. Also, because there are very few participants who receive more than 5 contact attempts, and these participants provide considerable weight in the repeated attempts model and might be unusual and influential, an analysis was performed omitting these participants.

Finally the number of telephone calls received was added as a covariate in (5) when *m* = 11. This allows the probability of the success of the email attempt to depend on the number of failed telephone calls.

In total this resulted in nine further fitted models and the estimates of *β*_2_ are shown in Table 7. Most of the estimates are similar in sign, magnitude and standard error. The two that differ in sign to the rest (from the analyses restricted to male and younger participants) are less well identified. This is reasonable because there are fewer male participants and younger participants are less likely to provide outcome data. Furthermore, these negative point estimates point in the opposite direction to “missing=smoking” and the impression from Table 7 is that none of the models fitted support this assumption.

**Table 7:** Further estimates of _*β*2 _from repeated attempts modelling

**Covariates included**	**Participants included**	_***β*****2**_
Smoking related	All	0.176 (0.228)
Non-Smoking related	All	0.258 (0.217)
None	All	0.245 (0.220)
All	Younger (36 and under)	-0.013 (0.606)
All	Older (37 and over)	0.333 (0.258)
All (except sex)	Men	-0.302 (0.738)
All (except sex)	Women	0.359 (0.240)
All	Those who receive 5 calls or less	0.296 (0.165)
All plus the email attempt depends on the number of failed telephone calls	All	0.217 (0.221)

Estimates of *β*_2_ are shown, with standard errors in parentheses, when omitting particular combinations of covariates and participants.

## Conclusions

We have developed two statistical models and have explored the missing data model using the empirical evidence from the iQuit trial. In particular we found strong evidence that the participant’s age is a good predictor in this. The evidence that the trial outcome itself is important in this model is much weaker. This casts very considerable doubt on the “missing=smoking” assumption. This conclusion is also evident from an inspection of Table 3; one can imagine what would happen if the attempts to obtain outcome data were ceased after fewer attempts. Some nonsmokers in Table 3 would then be lost to follow up and designated as smokers in error by the “missing=smoking” assumption. Future methodological research could focus on methods for assessing the goodness of fit and other diagnostics for the repeated attempts model.

Perhaps our most important finding is that we estimate the probability not smoking in those failing to provide outcome data to be between 0.14 and 0.19. This excludes both the “missing=smoking” assumption and the MAR analysis that makes makes no use of the baseline covariates (22% of participants who provide outcome data abstained from smoking). This finding, in conjunction with the arguments of Nelson *et al.*[9] and Barnes *et al.*[10], provide a case for “missing=smoking” analyses to be abandoned altogether. However the MAR assumption seems to be a good option, provided that suitable covariates are collected and included in the model.

We do not show parameter estimates of the outcome model (1) because we do not wish to distract the reader from the investigation of the missing data mechanism, which provides our focus. However when fitting this outcome model using maximum likelihood, in conjunction with either (2) or (5), all parameter estimates are obtained simultaneously. Hence parameter estimates of model (1) could be also presented such as the treatment effect, which is usually the parameter of primary interest.

The suspicion that participants with more educational qualifications may be more likely to respond to an email reminds us that the variables that are important in the missing data model are likely to be context specific, and can be anticipated to depend on the nature of the trial and how data are collected. For example, if email was the primary method for obtaining response data then, if correct, this suspicion suggests that qualifications would be a crucially important variable to consider when modelling the missing data. Trialists therefore should not take our investigation as a definitive statement of which variables are important in smoking cessation trials in full generality, but our results suggest that both smoking and non-smoking related variables can play a role in this. We therefore recommend that, if additional variables are to be incorporated into the analysis to make the MAR assumption more plausible, trialists should consider both kinds of variables, and also any other variables that they think may explain why their data are missing. A rich set of baseline, and possibly auxiliary post randomisation, variables should be collected for this purpose.

Even if the many such variables are collected and incorporated in the analysis then the possibility that the outcome itself may play a role persists, as epitomised by the “missing=smoking” assumption. However this requires MNAR modelling and the approaches used here, although suitable for our special investigations, are perhaps too computationally intensive for more routine use. We are therefore developing a simpler MNAR modelling approach, where “missing=smoking”, MAR and Last Observation Carried Forward analyses (LOCF [8, p. 45]) are embedded into a much wider class of models. Hence the implications of many possibilities for the treatment effect can be quickly and easily assessed. Despite the computational power that is now available, the trade-off between sophisticated methodology and computationally straightforward methods remains, so we hope that this will make MNAR modelling more accessible to applied researchers and that they will be inspired to attempt this.

## Appendix

We continue to let *R*_*i*_ = 1 denote that the *i*th participant provides outcome data and we let the binary variable *E*_*i*_ denote whether or not an attempt by email was made, where *E*_*i*_ = 1 if this was made and *E*_*i*_ = 0 otherwise. We let *t*_*i*_ equal the number of *telephone* call attempts to obtain outcome data. The log-likelihood of the data is given by *L*( *α*_0_, *α*_1_, *β*_0_, *β*_1_, *β*_2_)=

(8) $$\begin{array}{lll} & \overset{1758}{\underset{i=1}{\sum }}E_{i}R_{i}\log\left\{P(Y_{i}=y_{i}|x_{i})P(R_{i,11}^{\prime }=1|Y_{i}=y_{i},x_{i})\right)\; & \;\\ & \left(\;\;\;\times \overset{t_{i}}{\underset{j=1}{\prod }}P(R_{i,j}^{\prime }=0|Y_{i}=y_{i},x_{i})\right\}+\; & \;\\ & \overset{1758}{\underset{i=1}{\sum }}(1-E_{i})R_{i}\log\left\{P(Y_{i}=y_{i}|x_{i})P(R_{i,t_{i}}^{\prime }=1|Y_{i}=y_{i},x_{i})\right)\; & \;\\ & \left(\;\;\;\;\;\;\times \overset{t_{i}-1}{\underset{j=1}{\prod }}P(R_{i,j}^{\prime }=0|Y_{i}=y_{i},x_{i})\right\}+\; & \;\\ & \overset{1758}{\underset{i=1}{\sum }}E_{i}(1-R_{i})\log\left\{\overset{1}{\underset{y_{i}=0}{\sum }}P(Y_{i}=y_{i}|x_{i})P(\overset{\prime }{\underset{i,11}{R}}=0|Y_{i}=y_{i},x_{i})\right)\; & \;\\ & \;\;\;\;\;\;\left(\times \overset{t_{i}}{\underset{j=1}{\prod }}P(R_{i,j}^{\prime }=0|Y_{i}=y_{i},x_{i})\right\}+\; & \;\\ & \overset{1758}{\underset{i=1}{\sum }}(1-E_{i})(1-R_{i})\log\left\{\overset{1}{\underset{y_{i}=0}{\sum }}P(Y_{i}=y_{i}|x_{i})\right)\; & \;\\ & \;\;\;\;\;\;\left(\times \overset{t_{i}}{\underset{j=1}{\prod }}P(R_{i,j}^{\prime }=0|Y_{i}=y_{i},x_{i})\right\}\; & \; \end{array}$$

where the probabilities necessary to compute this likelihood are evaluated in terms of the *α* and *β* parameters from equations (1) and (5). Empty products in this likelihood are defined to be one.

## Competing interests

The authors declare that they have no competing interest.

## Authors’ contributions

DJ performed the statistical analyses and all authors contributed to the writing of the paper. All authors read and approved the final manuscript.

## Pre-publication history

The pre-publication history for this paper can be accessed here:

http://www.biomedcentral.com/1471-2288/12/157/prepub
